# Current trends and intricacies in the management of HIV-associated pulmonary tuberculosis

**DOI:** 10.1186/s12981-016-0118-7

**Published:** 2016-09-26

**Authors:** Narendran Gopalan, Padmapriyadarsini Chandrasekaran, Soumya Swaminathan, Srikanth Tripathy

**Affiliations:** Division of HIV, National Institute for Research in Tuberculosis (Formerly Tuberculosis Research Centre), No. 1, Mayor Sathyamoorthy Road, Chetpet, Chennai, 600 031 India

**Keywords:** HIV, TB, ATT, ART, IRIS, MDR-TB, IPT

## Abstract

Human immunodeficiency virus (HIV) epidemic has undoubtedly increased the incidence of tuberculosis (TB) globally, posing a formidable global health challenge affecting 1.2 million cases. Pulmonary TB assumes utmost significance in the programmatic perspective as it is readily transmissible as well as easily diagnosable. HIV complicates every aspect of pulmonary tuberculosis from diagnosis to treatment, demanding a different approach to effectively tackle both the diseases. In order to control these converging epidemics, it is important to diagnose early, initiate appropriate therapy for both infections, prevent transmission and administer preventive therapy. Liquid culture methods and nucleic acid amplification tests for TB confirmation have replaced conventional solid media, enabling quicker and simultaneous detection of mycobacterium and its drug sensitivity profile Unique problems posed by the syndemic include Acquired rifampicin resistance, drug–drug interactions, malabsorption of drugs and immune reconstitution inflammatory syndrome or paradoxical reaction that complicate dual and concomitant therapy. While the antiretroviral therapy armamentarium is constantly reinforced by discovery of newer and safer drugs every year, only a few drugs for anti tuberculosis treatment have successfully emerged. These include bedaquiline, delamanid and pretomanid which have entered phase III B trials and are also available through conditional access national programmes. The current guidelines by WHO to start Antiretroviral therapy irrespective of CD4+ cell count based on benefits cited by recent trials could go a long way in preventing various complications caused by the deadly duo. This review provides a consolidated gist of the advancements, concepts and updates that have emerged in the management of HIV-associated pulmonary TB for maximizing efficacy, offering latest solutions for tackling drug–drug interactions and remedial measures for immune reconstitution inflammatory syndrome.

## Background

HIV and tuberculosis (TB) have always been faithful comrades facilitating each other in spreading across the globe. According to recent World Health Organization (WHO) estimates, 9.6 million cases of tuberculosis (TB) occurred all over the world with 12 % (1.2 million) being co-infected with human immunodeficiency virus (HIV); with 37 % of these new TB cases going undiagnosed [1]. In the year 2014, an estimated 1.5 million had died due to TB with a quarter of them caused by HIV. Pulmonary TB is the commonest form of TB even in HIV even though it is more frequently associated with dissemination locally and systemically, when the two infections coexist. Therefore, early diagnosis and prevention of pulmonary TB, with suitable chemoprophylaxis have become key components towards achieving the end TB strategy [1].

Despite wide spread scale up of antiretroviral therapy (ART), only about one-third (392,000 or 77 % of the notified TB cases known to be HIV infected) were put on antiretroviral therapy (ART). The aim should be to combine the ideal anti-tuberculosis treatment (ATT) with mutually compatible highly active antiretroviral therapy (HAART) combinations to maximize efficacy, avoiding drug–drug interaction. When ART is initiated in HIV infected subjects, a major complication that could arise is the onset of ART related immune reconstitution inflammatory syndrome or IRIS, requiring, early detection and prompt treatment. These important aspects pertaining to diagnosis and treatment of this deadly duo is being elaborated in the following paragraphs.

## Diagnosis of tuberculosis in HIV infected

### Latent TB infection

HIV infection is one important factor for progression to TB disease, mandating meticulous screening and treatment for latent *M. tuberculosis* infection especially in TB prevalent countries.

### Diagnosed of latent TB infection

1. *Tuberculin skin test (TST)* Targeted tuberculin testing for LTBI forms a strategic component of TB control identifying high risk population prone for developing TB [2]. Studies have shown that TST-positive patients benefit more from IPT than those who are TST negative [3]. Anergy, improper cold chain maintenance can give rise to false negative results in HIV [2]. Considering these limitations in resource limited set-ups, World Health Organization’s Guidelines Group strongly recommends IPT irrespective of TST for people living with HIV [3].

2. *TB MPB*-*64 skin patch test* MPB-64 is a specific mycobacterial antigen secreted by *M. tuberculosis*, *M. bovis* and some strains of *M. bovis* used in this patch. This test is simple, non-invasive, does not require a laboratory or highly skilled personnel, unaffected by anergy in HIV-infected individuals and becomes positive in 3–4 days after patch application on skin, and induration on skin lasts for a week. In a study in Manila, Philippines the sensitivity of the transdermal Patch was 87.8 %, with an efficacy of 92.9 % and a specificity of 100 % [4].

3. *IFN*-*γ release assays (IGRA’s)* These in vitro blood assays based on IFN**-γ** production from sensitized T cells TB antigens like early secretory antigenic target 6 (ESAT 6) and culture filtrate protein 10 (CFP 10), are commercially available as QuantiFERON-TB QFT), Enhanced QuantiFERON-TB Gold assay and ELISPOT format, T-SPOT-TB assay] [5].

A study of asymptomatic adults from South Africa, a country with a high prevalence of co-infection found that the rates of positive ELISPOT and ELISA results did not vary significantly by HIV status compared to TST [6]. Due to the requirement of a good laboratory infrastructure and costs, the WHO’s Guidelines Group does not recommend IGRA to screen people living with HIV for eligibility to receive IPT, as IGRA does not spell out who will benefit most from IPT [3].

4. *Blood biomarkers* Studies are underway using host RNA gene expression from whole blood that allows for identification of prospective high risk individuals who can potentially progress to active tuberculosis disease [7].

### Diagnosis of active TB disease

High clinical suspicion is required in diagnosing early TB disease especially in the context of advanced HIV due to paucity of classical symptoms. WHO guidelines on systematic screening for active pulmonary TB using syndromic evaluation, with active case finding serves a dual purpose, channelizing individuals for either chemoprophylaxis or for prompt initiation of treatment [8–10]. This simplified syndromic questionnaire of three symptoms namely cough, fever and night sweats had been effectively used to diagnose or rule out TB in a study from South East Asia [11].

#### a. Imaging techniques

**Fig. 1:**
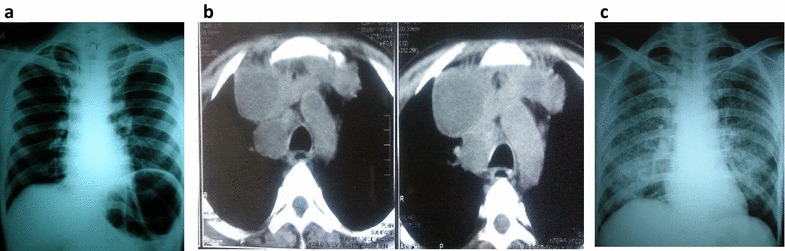
Varying manifestations of TB in HIV. **a** Apparently normal CXR. **b** Caeseating mediastinal adenitis typical of TB. **c** Miliary TB

Adding a chest X-ray or a CT scan to symptom screening not only increases the detection rate but the cost as well. The spectrum of radiographic manifestation of pulmonary TB is dependent on the relative level of HIV-related immunodeficiency and varies from normal chest X-ray (CXR) to full blown miliary TB [12, 13] (Fig. 1). A study to evaluate the utility of initial CXR in the diagnostic algorithm for symptomatic HIV-infected patients with negative sputum smears, found that basing a diagnosis of pulmonary TB on initial CXR leads to over diagnosis of TB. CXR had a sensitivity and specificity of 72 and 57 %, respectively, with positive predictive value of 21 % and negative predictive value of 93 % in diagnosing PTB [14]. In addition routine abdominal ultrasonography, followed by abdominal CT scan in inconclusive cases, significantly increased the detection of abdominal TB in patients with advanced immunodeficiency [15].

#### b) Microbiology

1. *Sputum Smear microscopy* The most frequent and conventional method of pulmonary TB detection in HIV co-infected persons involves examination of at least two sputum specimens, including one early-morning specimen, for acid-fast bacilli (AFB) [16]. Though the sensitivity of sputum microscopy in HIV infection is low (43–51 %), it has the advantage of being inexpensive, relatively rapid to perform, and specific in most settings. Fluorescence microscopy, light emitting diode microscopy, alternative specimen processing methods, such as concentration, bleach sedimentation and same-day sputum collection (front loading) strategies increase the sensitivity of sputum microscopy from 59 to 93 % in detecting pulmonary TB in HIV co-infected persons [17–19].

2. *Culture of Mycobacterium tuberculosis (M.Tb)* Mycobacterial culture still remains the gold standard for TB diagnosis. The inherent weaknesses of smear microscopy to determine viability, drug resistance and species identification has made culture test indispensable. HIV co-infection does not affect the yield of mycobacterial culture, but its growth on traditional solid medium like the Middlebrooks or Lowenstein-Jenson medium is rather slow requiring up to 6–8 weeks. Repeated sputum examinations for culture up to three specimens increases the yield by 10–17 % [16].

With increased sensitivity and reduced delays, automated liquid culture systems have replaced solid cultures, increasing case detection by 10 % with quicker TB diagnosis (detecting mycobacterial growth within 1–2 weeks), resulting in prompt treatment initiation. Methods currently in practice include Mycobacteria Growth Indicator Tube (MGIT) 960 [Becton–Dickinson Diagnostic Instruments Systems] using fluorescent sensors, MB/BacT system (Organon Teknika) using colorimetric sensors, ESP culture system II (Difco Laboratories, USA) using pressure sensors, or redox reagents, such as Alamar blue [20–22]. Among HIV-TB co-infected patients, MGIT was found to be more sensitive than culture and microscopy [23]. Other available techniques include bacteriophage based assays (FASTPlaqueTB), Luciferase reporter phage based test and Microscopic observation drug susceptibility (MODS) assays. The latter is a low cost non-commercial method for early detection of micro colonies and drug resistance [24, 25].

3. *Molecular techniques* Nucleic acid amplification tests (NAAT), are designed to amplify nucleic acid regions specific for *M. tuberculosis* complex and can be used directly on the clinical samples. NAAT yield rapid results, are highly specific with improved sensitivity compared to smear microscopy. These techniques can detect specific mutations, thus providing information on drug sensitivity as well.

Line probe assays (LPA), endorsed by the WHO in 2008 for molecular detection of drug resistance uses a PCR/hybridization technique to distinguish members of the *M.Tb* complex and simultaneously identifies drug-resistant strains by detecting the most common single nucleotide polymorphisms associated with resistance [25]. LPAs are highly sensitive (≥97 %) and specific (≥99 %) for the detection of rifampicin resistance, alone or in combination with isoniazid (sensitivity ≥90 %; specificity ≥99 %), on isolates of *M. tuberculosis* but it use is restricted to smear-positive sputum specimens only [26].

Xpert-MTB rif (GeneXpert) has currently taken the center stage, providing results within 2 h, with an increase in case detection rate of 45 % compared to smear microscopy and can be used in smear negative patients also [27]. It is a TB-specific automated, cartridge-based nucleic acid amplification assay, endorsed by WHO, for the rapid diagnosis of TB as well as early detection of rifampicin resistance among HIV-infected individuals among presumptive TB patients [27]. Xpert—Ultra is an improved version that has been equated to a liquid culture in its ability to detect Tuberculosis with an improvised specificity to detect MT. TB as well as rifampicin resistance even among smear negative patients with HIV where the conventional Xpert—MTB rif has a lesser yield [28]. Loop-mediated isothermal amplification and Fluorescence in situ hybridization using peptide nucleic acid probes, are other rapid and simplified molecular techniques using NAAT platform used to diagnose Mycobacterial infection, with high sensitivity and specificity [29, 30].

4. *Serological diagnosis of tuberculosis* Serological tests have no role to play for detecting active TB disease either pulmonary or extra pulmonary TB, and have been banned by WHO [31].

#### Other diagnostic techniques

Capture ELISA test is available to detect lipoarabinomannan (LAM) in urine specimens. Among hospitalized HIV-infected patients with suspected TB in Zimbabwe, the sensitivity of LAM was found to be significantly higher than that of Sputum smear microscopy, especially in advanced HIV with lower CD4 counts [32]. Bedside LAM-guided initiation of ATT for HIV-infected hospitalized patients with suspected TB was associated with a reduced 8-week mortality rate in a pragmatic, randomized, multi-centric trial in Africa [33]. WHO has now recommended the detection of LAM, in urine specimens in immunosuppressed HIV-infected patients as an adjunctive means of rapidly diagnosing active TB disease [34].

In-spite of all these newer TB diagnostic technologies, including Urinary LAM and Gene Xpert, clinical suspicion and thorough screening of TB in HIV still plays a vital role.

### Preventive therapy for TB in HIV infection

As the risk of TB developing in HIV infected individuals is 5–10 % every year, current WHO guidelines recommend screening of all HIV-infected individuals for TB (intensified case finding), and if found to be un-infected, receive isoniazid preventive therapy (IPT) for a 6 months, irrespective of TST status [35, 36].

Various randomized controlled clinical trials on different durations and multiple drug regimens have yielded varying results (Table 1).

**Table 1:** Summary of characteristics and conclusion of randomised controlled clinical trials using various regimens for TB prophylaxis in HIV

Author	Cohort characteristics	Median CD4	Study design used in RCT	No. enrolled	Drugs used with dosages and duration	Duration of follow up	TB breakdown	Conclusion
Samandari et al. [53]	HIV infected With ART (47 %) if CD4 <200 cells/mm^3^	297	Double blind placebo controlled	1995 patients	Arm A 6 months INH 300 mg daily +30 months placebo	36 months	Arm A 1.26 % Arm B-0.74 %	36 months INH was more effective but with greater toxicity
					Arm B 6 months INH 300 mg daily +30 months INH daily			
Swaminathan et al. [54]	HIV infected With/without ART	320	Open labelled	683 patients	Arm A 36 months of INH 300 mg daily	36 months	Arm A 1.6/100py Arm B-2.4/100py	Statistically similar efficacy and toxicity with 6EH7 and 36 INH. Emergence of resistance was 0.8 %
					Arm B-6 months of INH 300 mg and Ethambutol 800 mg daily			
Martinson et al. [55]	HIV infected without ART and TST positive	484	Open labelled	1148 patients	Arm A Rifapentine (900 mg) plus INH (900 mg) weekly for 12 weeks, Arm B Rifampin (600 mg) plus INH (900 mg) twice weekly for 12 weeks, Arm C INH (300 mg) daily for up to 6 years (continuous isoniazid) Arm D INH (300 mg) daily for 6 months (control group).	Not specified	Arm A-3.1/100py Arm-B 2.9/100py Arm-C 2.7/100py Arm-D-3.6/100py	All regimens had equal efficacy w.r.t 6 months INH with toxicity more in the 3 years regimen. Emergence of resistance was 3.4 %
Sterling et al. [56] TBTC Study 26/ACTG 5259 [56]	HIV infected with 30 % on ART or close contact of TB cases	500	Open labelled	399 patients	Arm A 3 months of 900 mg (max) INH and 600–900 rifapentine once weekly Arm B 9 months of INH 300 mg daily	33 months	Arm A-0.39/100py Arm-B 1.25/100py	Both regimens had equivocal efficacy but more toxicity in 9 months of INH

A Cochrane systematic review of 12 trials, published in 2010 among 8578 patients demonstrated that IPT reduced the risk of active TB by 64 % among TST positive HIV-infected participants, but only by 14 % among TST negative individuals [37]. The key message from the review was that efficacy was similar regardless of drug type, regimen or duration compared to INH monotherapy [37]. CDC also recommended a 12-weekly regimen of INH and rifapentine given as DOT as an equal alternative to 9-months of daily self-supervised INH for treating LTBI in otherwise healthy patients aged ≥12 years and advised against use of rifampicin and pyrazinamide for 2 months as prophylaxis [38, 39]. Despite evidences in favour of preventive therapy, adoption of TB preventive therapy in clinical practice has been pretty slow, primarily due to the difficulty in ruling out active TB in HIV infection, apart from reluctance on part of policy makers and program implementers. Getahun et al. [40] suggested IPT be taken over by the HIV intervention programme for an effective implementation after thorough screening for TB.

#### Patient selection for IPT

In 2011, the WHO released simplified guidelines for IPT, using the clinical algorithm of any cough, night sweats, weight loss and/or fever. Similarly, house hold contacts of sputum smear positive pulmonary TB cases, especially children, are a good entry point for screening for TB and delivery of IPT [36]. A community wide survey among South African mine workers found that majority (approximately 80 %) were eligible for IPT [41]. Due to low specificity of symptom screening, C-reactive protein (CRP) that has a higher specificity for active TB has been incorporated into the diagnostic algorithm. A point-of-care CRP could facilitate rapid initiation of IPT in HIV patients without active TB [42, 43].

#### IPT with antiretroviral therapy (ART)

Mortality within the first 6 months after initiating ART has been attributed to TB in most resource-limited settings. Protection against TB is further optimized when IPT is combined with ART. Synergistic protection, with greater than 50 % reduction in TB rates, was found in patients who received both IPT and ART than protection afforded by either treatment alone [44, 45]. However, empirical TB treatment in situations where it is difficult to diagnose subclinical TB like advanced HIV disease may not be of much benefit in reducing mortality [46].

#### Cost-effectiveness of IPT

A randomized trial of HIV infected adults from India showed that IPT, 6EH increased life expectancy by 0.8 months incurring $100 per individual while 36 months of INH extended life expectancy by 0.2 months with an additional per person cost of $55 [47]. In addition, antepartum IPT for HIV-infected women, irrespective of CD4 count and TST status, was shown to have greater health benefits and more cost-effective than no IPT or TST-driven IPT in a study from India [48].

Brazil demonstrated that training health care workers to screen HIV-infected adults with positive TST and providing IPT to those with latent TB infection was cost-effective relative to the Brazilian GDP per capita [49]. Similarly, the trial from United States using 12-dose of weekly rifapentine plus isoniazid administered as directly observed treatment, also proved to be a cost-effective alternative to 9 months of daily self-administered isoniazid [50]. A decision-analysis model to study the cost-effectiveness of IPT in Botswana revealed that treating PLHIV who have positive TST with 36-month IPT was more cost effective for reducing both TB and death compared with providing IPT without a TST, providing only 6-month IPT, or expanding ART eligibility without IPT [51].

#### Drug resistance and IPT

A systematic review, assessing the effect of IPT on the risk of INH resistant TB, reported that IPT increases the risk of INH resistance by 1.45 times, whilst it was not significant (Relative risk 1.45; 95 % confidence interval 0.85–2.47) [52]. Though the study had a small sample size and analysis was restricted by incomplete testing of isolates, the relative risk showed a minimal increase in INH resistant TB. However, clinical trials of IPT in HIV-infected patients in Botswana, India and South Africa with more defined cohorts did not show any increased risk of INH resistance amongst patients given IPT [53–56].

### Summary

Latent TB is still a difficult diagnosis to make in the wake of HIV coinfection. Advances in molecular technology have partially replaced conventional cultures helping in prompt diagnosis of active TB yielding sensitivity results at the earliest. TB should be meticulously screened and INH preventive therapy for 6 months or any other equivalent regimen should be started for TB-free HIV-infected individuals irrespective of Tuberculin Skin Testing. There is a need to update the knowledge and awareness of INH Preventive Therapy among health care workers and policy makers for rapid implementation and reap the benefits as it can be easily coupled and monitored with ART initiation.

## Treatment for HIV-associated pulmonary TB

### ATT drugs to be used

Management of pulmonary TB in HIV demands meticulous monitoring as it is complicated by smear negativity and atypical presentation, emergence of drug resistance, Immune reconstitution inflammatory syndrome, drug–drug interaction and increased pill burden [57]. The Standards of TB care in India (STCI) recommend a four drug regimen consisting of Isoniazid (INH), rifampicin, pyrazinamide and ethambutol given during in the intensive phase of 2 months followed by INH and rifampicin with/without ethambutol (optional) in the next 4 months for treatment naive patients along with high dose pyridoxine and cotrimaxazole in HIV-TB coinfection [58]. HIV infection favours mycobacteremia and tissue invasion resulting in abundance of intracellular and intermittently dividing bacilli, making rifampicin indispensable in HIV associated TB [59]. This is despite therapeutic interactions with other concomitant medications notably HAART. Non-rifampicin regimens in HIV have been associated with inferior outcomes coupled with longer duration of therapy [60, 61]. The addition of quinolones in the intensive phase does not increase cure rate any further [62]. Interestingly, the sputum conversion rate is faster in HIV-PTB coinfection with effective ATT [63]. Studies comparing TB treatment outcomes between HIV infected and uninfected individuals have obviously shown better results in the latter group [64].

Empirical ATT even in advanced HIV has no definitive role to play. The A5274 study clearly demonstrated that even in a high TB burden population of 1368 participants with advanced HIV (CD4 < 50 cells/mm^3^), there was no benefit of empiric TB therapy over IPT when mortality rates at 48 weeks was compared [65].

#### Duration of TB therapy

TB treatment duration is not influenced or confounded by HIV infection currently being 6 months for Pulmonary and extended in severe forms of extra pulmonary TB like bone and neurological TB. Centre for Disease Control, Atlanta recommends extension of ATT beyond 6 months in HIV-coinfected pulmonary TB patients in specific instances like delayed sputum conversion or poor clinical improvement with/without evidence of dissemination, low CD4 count at nadir and presence of cavitation [66]. Swaminathan et al. [67] compared a 6 months standard intermittent therapy with a 9 months regimen (an extended continuation phase of 3 months) in the pre-HAART era among HIV infected pulmonary TB patients, and found that the favourable outcomes, failures at the end of treatment, adverse events and mortality (during treatment and follow-up of 24 months) to TB were similar. However, there was a significant reduction in bacteriologically confirmed recurrences when a longer regimen was used. The meta-analysis by Menzies et al. [68] demonstrated that extended treatment beyond 6 months (to 8 months) did not appreciably increase the efficacy further, justifying the current duration of 6 months. The same meta-analysis found only a single study having a higher risk of relapse with extensive cavitary disease demanding 8 months treatment. Hence, the authors, concluded that high risk identification would not be easy in the public health perspective that makes generalized extension of regimen in all cases unnecessary [68].

#### Dosing schedule

The legacy of intermittent regimens in India dates back to 1964 when Tuberculosis Research Centre used streptomycin and INH administered twice weekly, yielding a cure rate of 94 % and a relapse rate of 8 % [69]. The popular attributes of intermittent regimens were operational feasibility, lesser cost, fewer side effects and ease of supervision. The scientific basis for intermittent usage of ATT is based on post antibiotic effect of ATT or lag period [70]. Mycobacterium undergoes a lag phase in its growth pattern due to the influence of the post antibiotic effect, when bacilli fail to grow for a period of a few days even after removal of the exposed drug i.e. ATT [70]. However, newer hypotheses challenge this approach. It has been postulated that the ratio of peak concentration to minimum inhibitory concentration better correlates with the post antibiotic effect and contributes to suppression of resistance than lag phase. The authors further hypothesized that asynchrony between the metabolism of these intermittently growing bacilli and the day of drug administration could facilitate emergence of drug resistant mutants [71].

Swaminathan et al. [67] study showed a higher incidence of acquired rifampicin resistance (ARR) among failures, using thrice weekly rifampicin in HIV-PTB, which was unaltered by the duration of the regimen used. Interestingly, the meta-analysis by Menzies et al. [68] did not find intermittent ATT dosing to be a cause of poorer outcomes unless the frequency of dosing went down to below thrice a week, but had only 2 % of patient in the meta-analysis being co-infected. However, the meta-analysis by Faiz khan et al. showed that the use of intermittent regimen in HIV, especially in the intensive phase, increased the risk of failures, relapses and emergence of drug resistance which further intensifies in the wake of baseline H-resistance [72]. The study by Li et al. [73] showed that a daily intensive phase of ATT followed by an intermittent continuation phase served as a useful alternative to a fully daily regimen. The updated review by Faiz khan et al. [74] recommended use of a daily regimen throughout and extension of TB regimen to 8 months, but suggested further evaluation of data to make categorical conclusions. Recently, there has been a paradigm shift in approach to TB treatment among HIV in India, with the STCI recommending daily regimen for all HIV co-infected patients with ethambutol reinforced in the continuation phase (2EHRZ7/4HRE7) [58]. The only randomized trial of head to head comparison of daily vs thrice weekly ATT regimen in HIV patients with newly diagnosed rifampicin sensitive pulmonary TB, started on timely ART, showed a higher cure rate with daily compared to thrice weekly ATT (90 vs 76 %) but at the expense of higher incidence of hepatoxicity [75].

#### Acquired rifamycin resistance (ARR)—a unique phenomenon complicating HIV-associated pulmonary TB

The greater percentage of persistors and bacillary mutants in an immunocompromised environment (of HIV coinfection) facilitates and favours emergence of drug resistance to ATT notably rifampicin [76]. Acquired rifamycin resistance (ARR) is the emergence of resistance (defined as MIC > 128 µg/ml) to rifamycin among patients whose pretreatment isolates were sensitive. ARR is a rarity in HIV seronegative individuals with PTB. In a cohort of 1435 HIV sero-negative patients with drug susceptible PTB enrolled in various trials at the Tuberculosis Research Centre, Chennai, only 4 patients developed rifampicin resistance irrespective of the dosing schedule [77].

On the contrary, ARR in the HIV co-infected had been reported with all types of rifamycin regimens used intermittently, be it once weekly rifapentine [78], twice weekly rifabutin [79, 80] or thrice weekly rifampicin [67]. Advanced stage of HIV, absence of HAART, extensive and/or disseminated TB, initial H-resistance, and suboptimal drug concentrations due to malabsorption are contributing factors for ARR. Increased tissue bacillary load in HIV, coupled with defective clearance attributed to subdued immune apparatus leads to selection of genomic mutants resistant to rifampicin, which is much more pronounced in the face of baseline INH resistance [80–83]. The study by Narendran et al. [84] showed that HIV and INH resistance at baseline were significant risk factors associated with ARR and HAART only reduced the frequency but did not offset the trend. The NCT 00933790 trial evaluating the incidence of failures and ARR among co-infected with different dosing schedules of ATT and timely HAART showed that ARR occurred only with intermittent dosing [75].

### Recurrences

TB recurrences can be either due to endogenous re-activation or exogenous re-infection, the relative proportions depending on the background incidence of TB, level of immune suppression, length of rifampicin-containing ATT and adherence to treatment [79, 85]. The proportion of recurrences due to re-infection is more frequent in HIV positive individuals especially in countries with a higher TB burden than HIV-seronegative individuals with TB who have a true relapse [86].

### Persistence of smear positivity in HIV—are they true failures?

The decision making based on sputum smears alone can often be misleading and one such situation is the phenomenon of persistent sputum positivity despite regular treatment. A practical approach requires segregation of cases into patients with clinical improvement but smear positive or those who are truly failing therapy with persistence/recrudescence of clinical symptoms or both currently the national programme in many countries has scaled up to detect early drug resistance using line probe assay and Xpert-MTB Rif at the start of TB treatment that can simplify the problem [87]. Causes of true failures in HIV associated TB include emergence of ATT drug resistance, virological failure to ART, immunological discordance (lower CD4 with undetectable viral load) and malabsorption of drugs leading to cryptic non-adherence [79, 80, 82]. All of them may influence smear and culture conversion with the risk of subsequent failures or recurrences and transmission. Mal-absorption of ATT drugs may cause persistence of symptoms with delayed clearance of organisms, providing a favourable nidus for emergence of drug resistant mutants [81–83].

The second group of apparent failures comprises of patients with clinical improvement but still have sputum smears positive for AFB or have apparent deterioration. The differential diagnosis of the latter includes IRIS [88], transient resistance or “bacillary escape” [89] and non-tuberculous mycobacteria mimicking TB [90]. Atypical mycobacteria including *M*. *avium intracellulare, M. kansasi, M. fortitium* have been isolated more frequently in advanced stages of HIV disease when the CD4 is <100 cells/mm^3^ [90]. Nocardiosis may be misdiagnosed as TB due to its close resemblance to the latter both microscopically as well as clinically [91].

### Shortening TB treatment—a myth or a reality?

Whether a shorter TB regimen can work in the face of HIV coinfection still remains a mystery. The RIFAQUIN trial tested a combination of moxifloxacin replacing Isoniazid in the intensive phase along with rifampicin, pyrazinamide and ethambutol given daily in the 2 months intensive phase followed by high dose rifapentine (900/1200) for 2 months twice weekly or 4 months once weekly with moxifloxacin in the continuation phase and compared to a standard 6 months. The study failed to demonstrate non-inferiority of the 4 months regimen over the standard 6 months regimen. However, the 6 months regimen administered once weekly during the continuation phase showed comparable efficacy with better compliance and feasibility of implementation [92]. An important limitation in this study was that it enrolled only a quarter of HIV patients, with a relatively higher level of CD4 (above 200 cells/mm^3^) that limited its generalisability to advanced stages of HIV. The OFLOTUB study, that used gatifloxacin replacing ethambutol, also could not prove the 4 months regimen to be non-inferior to the 6 months regimen. These findings were more evident and obvious in the South African site which had half of their recruited patients being co-infected with HIV [93]. A regimen of PA_824 along with moxifloxacin and pyrazinamide, the STAND trial was being evaluated as a non-rifampicin, non-INH alternative that could prove useful in drug sensitive and MDR-TB, without significant drug interaction with ART. The study would resume enrolment shortly after being suspended temporarily [94]. A recent model had been developed that could predict efficacy of new regimens of varying durations, taking into account culture positive rate at 2 months and could spell out the recurrence rates to help investigators formulate shorter regimens with better efficacy [95].

### Summary

A standard four drug rifampicin containing regimen given daily for 6 months is still the ideal regimen for pulmonary TB in HIV despite frantic efforts globally to shorten TB treatment. Ethambutol can be added in the continuation phase. Empirical Antituberculosis therapy has no role to play. Acquired rifamycin resistance is a unique complication in HIV-TB. Patients failing therapy should not only be evaluate for ATT resistance but also efforts taken to rule out virological failure, malabsorption and screened simultaneously for non-tuberculous mycobacteria.

## Multidrug-resistant (MDR) and extensively drug-resistant (XDR) tuberculosis

Globally in 2014, an estimated 480,000 people developed multidrug-resistant TB (MDR-TB) with an estimated 39.5 % deaths and 9.7 % harboring extensively drug resistant TB (XDR-TB) [1]. India, China and Russia contribute about 60 % of global burden. While MDR-TB appears not to cause infection or disease more readily than drug susceptible TB in the HIV infected population, delayed diagnosis, inadequate initial treatment, and prolonged infectiousness contribute to increased attack rates among contacts, leading to high case fatality rates among patients [96].

Treatment for drug resistant-TB consists of at least 4–5 effective drugs. This includes a fluoroquinolone, a second line injectable agent (capreomycin, kanamycin or amikacin) and at least 2 agents from the remaining second line anti-TB drug classes [cycloserine, thionamides (ethionamide or prothionamide), linezolid, clofazimine] along with add on drugs like pyrazinamide, ethambutol, high dose INH, bedaquiline, delamanid, Amoxycillin clavulanate, para-amino salicylic acid selected preferentially in the order described above [97]. The intensive phase can be up to 8 months with a continuation phase without injectable up to 20 months depending on the therapeutic response. WHO had also come out with a shortened 9–12 month regimen based on the findings conducted by the International Union against Tuberculosis and Lung Diseases and Medical Research Council, United Kingdom, that consisted of moxifloxacin, clofazimine, prothionamide and pyrazinamide for 9 months reinforced with kanamycin and INH high dose in the first 4–5 months of intensive phase. This regimen, however, is approved for use in second line ATT naïve patients (<1 month of treatment) with DST pattern showing sensitivity to both quinolone and second line aminoglycoside at baseline, without extra-pulmonary or disseminated form of TB or pregnancy complicating TB [98].

Evidence for this regimen originated from the Bangladesh observational cohort study by Van Deun among MDR-TB patients which showed a relapse free cure rate of 84.5 % among 515 patients, but in a virtually HIV-free population [99]. The same regimen, tested in the francophone African countries, among 408 patients (that included HIV positive −22 %) showed a relapse free survival rate of 82.1 %. Although treatment success rates did not differ by HIV status among those who survived, the death rate was higher among HIV co-infected 18 % died, compared to 5 % in HIV-seronegative patients [100].

Prognosis of MDR-TB in HIV continues to be grave with a death rate of over 50 % once again highlighting the ardent need to reconstitute the fallen immunity by timely ART initiation [101]. Two additional factors which hinder TB control among drug resistant cases include nosocomial transmission and increased incidence of drug toxicity with frequent drug interruptions that could worsen the prognosis [102, 103]. Both the conditions can lead to amplification of drug resistance and need to be addressed urgently. Stringent airborne infection control measures need to be in place as an effective strategy and overcrowding in hospitals minimized [102]. One study, surprisingly, reported a toxicity of only 7 % among 2000 patients in a study in South Africa elucidating the fact that it is comparable to HIV seronegative group [104].

XDR-TB or extensively drug resistant defined as MDR-TB (resistance to INH and rifampicin) plus resistance to any fluoroquinolone and one of the second line anti-TB injectable agents (kanamycin, amikacin or capreomycin) constitutes a formidable medical challenge. Although the absence of rifampicin brings down the occurrence of drug–drug interactions significantly, therapy for XDR-TB is confounded by other adverse reactions like QTC prolongation, anemia, psychiatric effects, nephrotoxicity and gastrointestinal intolerance in addition to increased pill burden [103]. Treatment options are extremely limited and challenging with higher frequencies of adverse events and death [96]. In a recent study on long term follow-up of XDR-TB patients from South Africa, independent predictors of probability of net sputum culture conversion were no previous history of multidrug-resistant tuberculosis (p = 0.0007), use of clofazamine (p = 0.0069) with survival depending on culture conversion (p < 0.0001), treatment with clofazamine (p = 0.021) and ART in HIV (p = 0.003) [105].

### Newer TB drugs in the pipeline

Bedaquiline (BDQ), one of the newer drugs conditionally approved by the FDA recently, has been a useful adjunct in drug resistant-TB therapy, especially when choice is limited. It is a diarylquinoline derivative that inhibits the mycobacterial ATP synthetase [106]. It inhibits both actively replicating and non-replicating mycobacteria with no cross resistance to any of the first line drugs and quinolones. It takes 3–5 days for perception of its bactericidal effect and has an extremely long half-life of 4–5 months and has to be taken along with food. It is administered 400 mg daily for the first 2 weeks followed by 200 mg thrice weekly up to a total of 24 weeks. CYP3A4 is the major cytochrome isoenzyme that metabolizes bedaquiline to M2, that is mainly removed in the stools, with only 1–4 % excreted in urine [107]. Drug-drug interactions occur with CYP3A4 inducers (e.g., rifampicin reduced bedaquiline exposure by approximately 50 %, obviating co-administration). The most frequent suspected adverse reactions (>20.0 % of patients) during treatment with bedaquiline in the controlled trials (C208 stages 1 and 2) were nausea (35.3 %), arthralgia (29.4 %), headache (23.5 %), hyperuricemia (22.5 %), and vomiting (20.6 %) [106]. The magnitude of QTcF prolongation, one of the potential side effects of BDQ was greater in patients treated concomitantly with clofazimine but none of them ever developed torsade de pointes [108]. One novel way that is being evaluated in preclinical studies to counteract the effect of Bedaquiline causing QTC prolongation is by adding verapamil [109]. Delamanid (OPC67687) 100 mg, has also entered phase IIb and III trials. Follow up data from trial 204 which enrolled 481 MDR-TB patients showed a 74.5 % favourable response among patients using delamanid for 6 months or more, compared to 55 % with use of delamanid for less than 2 months [110]. PA-824, a nitroimidazole (along with moxifloxacin and pyrazinamide) proved efficacious and safe in the phase II b study showing a greater bactericidal activity compared to standard ATT and is used in the STAND trial [94, 111].

### Newer TB drugs and ART and drug–drug interactions and therapeutic implications

Bedaquiline concentration is increased with co-administered lopinavir/ritonavir by 22 % due to reduced clearance, while BDQ concentration is reduced by 50 % with efavirenz and these combinations are better avoided. Nevirapine has no interaction with BDQ and can be given safely [112]. Delamanid is expected not to have any clinically significant interaction with EFV, NVP or boosted PI and can be used concomitantly, as it neither induces nor inhibits the CYP 450 system. PA-824 concentrations are reduced by both EFV and lopinavir/ritonavir being 35 and 17 % respectively [113]. Newer derivatives of Linezolid in the pipeline are sutezolid, AZD 5487, radezolid and tedizolid, which are devoid of the myelosuppressive side effects of the linezolid, their predecessor [114].

## Antiretroviral treatment (ART) in HIV-PTB

### The influence of HIV and ART on TB outcomes

In the pre-HAART era, a number of studies have shown considerably lower cure rates, higher mortality and recurrence rates of TB after standard ATT in HIV co-infected patients compared to sero-negative individuals [115–117]. The study in Spain showed an efficacy of 43 % in HIV infected Vs 70 % in the HIV negative TB [115]. Nevertheless, the study by Chaisson et al. did not reveal the outcomes to be different based on HIV status (87 % in the uninfected vs 81 % among co-infected). The median CD4 count of the study cohort was 475 cells/mm^3^ suggesting that preservation of adequate immunity could potentially improve TB outcomes in HIV emphasizing the need for early ART initiation [116]. Treatment of TB alone in HIV-TB co-infected patients did not substantially increase CD4 count nor reduced the viral load [117]. This had prompted both the WHO [118] and the National AIDS Control Organisation, (NACO) India [119] to recommend ART initiation irrespective of CD4 in HIV-TB infection. The updated systematic review on TB treatment in HIV by Faiz khan et al. of which the authors had also contributed, showed substantial improvement in TB treatment outcomes, reduction in relapse rates, mortality and acquisition of drug resistance with early ART initiation [74]. Timely ART initiation undoubtedly improved TB outcomes. Comparing HIV-TB studies conducted at NIRT in the pre- and post-ART era, the TB outcomes were significantly better with HAART co-administration (favourable response of 93 vs. 83 %) with a reduction in all-cause mortality from 17 to 5 % [67, 120]. Pulmonary TB patients initiating ART in the intensive phase had a better TB outcome (adjusted odds ratio 1.83 (95 % CI 1.29–2.60) compared those not on ART from a study from Malawi [121]. A study from Thailand also reflected the same trends with 43 % mortality in the absence of ART compared to 7 % when co-administered with ART [122].

#### The ideal ART regimen for TB co-infected patients and newer options available

Tenofovir, emtricitabine/lamivudine along with efavirenz as a single pill once a day is most ideal [123]. That efavirenz can still maintain an adequate plasma despite co-administration with rifampicin makes it suitable for co-administration with rifampicin in HIV-PTB [124, 125]. While efavirenz dose (600 vs 800 mg) and concurrent rifampicin administration had less clinical impact [126], a polymorphism in the CYP2B6 gene (G–T mutation) resulted in significantly higher blood levels of the drug resulting in increased risk of neuro-psychiatric toxicity [127]. Trials have shown lower dose of EFZ (400 mg) to be therapeutically useful, with effective virological suppression but with a lesser toxicity (39 vs 48 %) at the same time compared to the standard 600 mg [128]. Use of nevirapine (available as generic fixed-drug combination) is not recommended routinely with rifampicin, unless there is a contraindication to efavirenz like pregnancy or psychiatric illness. However, nevirapine initiation should be without a “lead in” period, starting with 200 mg twice daily to maximize efficacy in the presence of rifampicin [120, 129]. Caution needs to be exercised in patients with a higher CD4 (>400 in males and above 250 in females) who are prone for fulminant hepatitis while using this strategy [123]. Triple NRTI regimens containing abacavir can be used alternatively in patients negative for HLA B57:01, but virological suppression is inferior especially when the viral load is high [130]. A quadruple NRTI regimen including tenofovir has been found to be as effective as EFV based regimen in trials of a smaller number [131]. Rifabutin offers greater flexibility than rifampicin and the modifications that are required in concomitant therapy is depicted in Table 2.

**Table 2:** Therapeutic modifications that are required while administering rifamycins and ART concomitantly [103, 177]

	Rifampicin (dosage-600 mg unless specified) interaction with ART modification and recommendation	Rifabutin (dosage-300 mg unless specified) interaction with ART modification and recommendation
Nucleoside reverse transcriptase inhibitors (NRTI’s)	No dose modification required Alternative regimens used only when NNRTI’s are contraindicated with VL < 100000copies/ml [103] Triple and quadruple NRTI regimens – caution to be exercised with triple regimen [178]	No dose modification required
Non-nucleoside reverse transcriptase inhibitors (NNRTI’s)		
Nevirapine	Reduced by 55 % [179–182] Not recommended routinely. If required., to avoid a lead in dose and start 200 mg BD [129]	No dose modification required as an alternative regimen with NVP
Efavirenz	Safe option, reduction only 20–25 % [183]. More dependent on CYP2B6 G516 G > T [184] Preferred with rifampicin	Increase rifabutin dosage to 450–600 mg, usually not recommended
Delavirdine	Not recommended	Not recommended
Etravarine	Reduction in NNRTI by 70–80 % [185]. Not recommended	Reduced by 35 % and Etravarine reduces rifabutin by 17 %. Same dose as rifabutin 300 mg [185]
Rilviprine	Reduction in NNRTI by 70–80 % [186]. Not recommended	
Doravirine	Not recommended	
Protease inhibitors		
Lopinavir/ritonavir	Not recommended	150 mg daily
Saquinavir All other PI’s [177] Amprenavir Indinavir	Not recommended Increase Indinavir to 1000 mg thrice a day.	300 mg daily 150 mg thrice weekly 150 mg daily
Super boosting [51]		
Lopinavir 400/ritonavir 400	Not recommended due to toxicity [187–190]	Super boosting not required for rifabutin
Double dosing [51]		
Lopinavir 800 mg/ritonavir 200 mg	Not recommended due to toxicity [187–190]	Double dosing not required for rifabutin
Integrase inhibitors		
Raltegravir [192, 193]	Reduced by 60 % [191] Increase dose of raltegravir to 800 mg BD [193] Caution to be exercised in patients with higher VL	400 mg BD of raltegravir
Dolutegravir [134]	Increase to 50 mg BD of dolutegravir	25 mg BD of dolutegravir
Elvitagravir/cobicistat	Not recommended	Not recommended as reduced by 67 % [194]
CCR5 Inhibitors		
Maraviroc	Not recommended	Not recommended

*CYP* cytochrome P450

Recently discovered drugs in the pipeline include tenofovir alafenamide (GS 7340), a prodrug of tenofovir, that gets converted at the site of lymphoid involvement and liver making it more potent, with greater tissue infiltration at a dosage ten times less than the conventional tenofovir and is currently evaluated in phase III trials in a fixed dose combination. There was significant improvement in estimated glomerular filtration rate, bone density and reduced proteinuria compared to conventional tenofovir disoproxil fumarate [132]. Dolutegravir has been shown to have a superior tolerability compared to darunavir in the Flamingo study [133]. The dosage of dolutegravir has to be doubled if concomitantly administered with rifampicin to 50 mg BD instead of the OD dosage (given with rifabutin) especially if given along with EFV [134]. Alternatively, dolutegravir 50 mg BD can also be combined with lamivudine and abacavir [123].

#### When to start ART after ATT initiation

Evidences from the START [135] and the TEMPRANO study [136] has made WHO recommend early initiation of ART, even in asymptomatic HIV individuals irrespective of CD4 so that Immune restoration is near complete with reduction in mortality due to both infective and non-infective causes with comparable toxicities. The START study which had 62 % of events attributed to TB showed that six participants in the immediate arm and 20 in the deferred arm of ART had broken down with TB favouring immediate ART initiation [135]. Early initiation of ART not only reduces mortality and morbidity due to HIV and TB with faster sputum conversion but also prevents IRIS secondarily [79]. The SAPIT trial observed 56 % lower mortality among patients who concomitantly started ART during TB treatment compared to those who subsequently started ART after ATT completion [137]. The important finding that can be deduced from the results of both the STRIDE [138] and SAPIT [139] trials is that while HIV-TB patients with a CD4 > 50 cells/mm^3^ need to start ART at the earliest with counselling on early recognition of signs and symptoms of IRIS and toxicity, a permissible delay of ART initiation among patients with higher CD4 up to 12 weeks after starting ATT helps in reducing drug interactions between rifampicin and NNRTIs, cumulative drug toxicities (especially hepatotoxicity), pill burden and the incidence of immune reconstitution inflammatory syndrome without any compromise on survival or predisposition to newer opportunistic infections [140].

The CAMELIA study enrolling 661 patients with a median follow-up of 25 months reflected similar findings that were deduced from patients in the lower range of CD4 in the SAPIT and STRIDES study. Early ART initiation by 2 weeks post ATT reduced mortality significantly compared to late initiation of ART at 8 weeks post ATT (18 vs 27 %, p = 0.0006). However, the risk of IRIS was 2.5 times more in the early ART group [141].

Current WHO and ART guidelines in India also recommend ART between 2 and 8 weeks of starting ATT on similar principles [119, 142].

Adverse reactions occur more often among HIV-infected patients with TB on concurrent medication than among TB without HIV (serious ADR—27 vs 13 %), occurring mostly in the first 2 months of treatment [143, 144]. Hepatoxicity is common due to shared metabolic pathways of anti-TB and antiretroviral drugs, alcoholism, co-infection with Hepatitis B and C, IRIS hepatitis and obstruction by nodes at the porta hepatitis all predispose to liver injury [88]. Most cases present with transaminitis which resolves when drugs are withheld. In drug sensitive TB, non-hepatotoxic drugs like streptomycin, ethambutol and ofloxacin should be substituted for TB treatment till liver functions return to normal, when all drugs can be re-introduced. Other adverse reactions include cutaneous and gastrointestinal symptoms and peripheral neuropathy which can occasionally be disabling [88]. Lactic Acidosis can present in various ways and needs periodic estimation [145].

Isolated hyperbilirubinemia is very common with use of atazanavir but is more of a cosmetic disfigurement that may stigmatise the patient. Zinc sulphate supplementation may be useful in management of ATV-related HBR in selected patients [146].

### Summary

The ideal co-administered ART regimen with ATT is a single pill of tenofovir (300 mg) along with emtricitabine/lamivudine (300 mg) and efavirenz (600 mg) initiated between 2 and 12 weeks after ATT initiation, provided the patients’ CD4 cell count is above 50 cells/mm^3^ with compromise on survival, (when there is no clinical compulsion). However, among patients with a CD4 of less than or equal to 50 cells/mm, immediate initiation of ART is the rule with proper counselling for IRIS and close monitoring for cumulative/additive toxicity that will help to maintain adherence

### Tuberculosis immune reconstitution inflammatory syndrome (TB-IRIS)

**Fig. 2:**
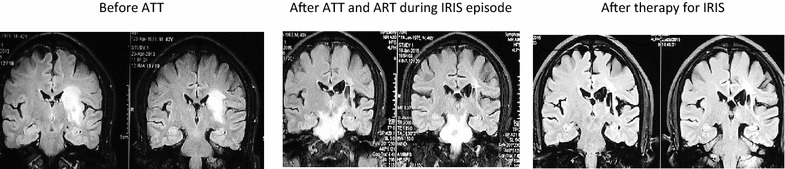
Pontine abscess as a manifestation of paradoxical TB-IRIS in a patients with HIV (& hemiparesis)

Tuberculosis immune reconstitution inflammatory syndrome (TB-IRIS) is the paradoxical worsening of symptoms and signs of TB after starting ART (rarely with ATT itself), despite a favorable immunological recovery and effective virological suppression [147]. Incidence ranges from 8–43 % [148]. The frequency and severity of IRIS depends on the degree of CD4 lymphocytopenia at nadir, the presence of other opportunistic infections and also the strategic location of the lesion. Figure 2 shows a pulmonary TB patient with HIV who presented with IRIS lesions in the brain after initiating ART [149, 150]. TB-IRIS is of two types (1) Paradoxical TB-IRIS that occurs in HIV-TB co-infected patients started on ATT and subsequently started on ART. Paradoxical IRIS is relatively easy to diagnose because of its biphasic pattern of initial improvement with ATT followed by a latter phase “paradoxical” deterioration after ART initiation. (2) Unmasking TB-IRIS or ANTIRETROVIRAL therapy (ART) associated TB occurs in an asymptomatic individual without a prior diagnosis of TB who starts developing symptoms with ART initiation. This could either be subclinical or undiagnosed TB [147, 151].

### Predictors of IRIS—risk factors

The most consistent risk factors are very low levels of CD4 cell count, CD4/CD8 ratio, hemoglobin, weight, presence of disseminated disease, shorter ATT-ART time interval, extra pulmonary foci and other opportunistic infections at the time of ART initiation [150–152]. Interleukin-6 and CRP were found to be reliable predictors of IRIS in a pure cohort of culture positive TB in HIV prospectively followed for IRIS occurrence in India. Evaluation of predictors from the CADRIS trial showed that baseline levels of vitamin D and higher D-dimer, Interferon gamma (IFN-γ), and sCD14 were independently associated with risk of IRIS [153]. TNFA-308*2 and IL-6174G gene polymorphisms have been implicated in TB-IRIS causation [154].

### Pathogenesis of TB-IRIS

Functional restoration of immunocompetent cells (CD4) causing a cytokine outburst with an overriding Th1 over Th2 response, apart from increase in number and their redistribution to the site of the lesion, is the primary mechanism of IRIS causation [155]. The majority of paradoxical IRIS cases occurs within the first 3 months after starting ART [156]. The T-regulatory cells or committed suppressors of the immune system are normally restored post ART but are functionally defective, tilting the tide towards an inflammatory reaction [157]. Recently, MMP-7, a tissue matrix metallo-proteinase, has also been implicated in TB-IRIS [158].

### Clinical features of TB IRIS

Fever with rigor or chills (resembling malaria) is the commonest and consistent symptom of TB-IRIS, with lymph node enlargement being the commonest manifestation [88]. Symptoms vary in severity from localized superficial lymphadenopathy and subcutaneous abscesses to severe forms like adult respiratory distress syndrome, meningitis, enlarging space occupying lesions like tuberculomas and viscus perforation, which can end fatally [151, 159]. Compressive features due to lymphadenopathy include stridor due to tracheal narrowing and superior vena caval (SVC) obstruction [160]. Patients with abdominal TB may present with pain and diarrhea. Other abdominal manifestations include hepatosplenomegaly, psoas abscesses, splenic micro abscesses, splenic rupture, epididymo-orchitis, uretric compression, and acute renal failure [159]. Osteomyelitis, sub-cutaneous abscesses and thromboembolic episodes have been reported [160]. Radiological worsening in pulmonary TB without symptoms or “cryptic IRIS” has been reported among TB patients alone after starting ATT in the pre HIV era also [161].

### Diagnosis of IRIS

It is important for physicians to remember that the onset of this syndrome is linked temporally to ART initiation, ART substitution (from I line to II line suppressing viremia) and ART interruption followed by re-initiation [88]. A strong suspicion and awareness of IRIS supplemented by the immunological tools of CD4 T cell count and viral load, after ruling out toxicity and drug resistance, will help clinch the diagnosis [162]. Initial work up of febrile episodes for infections endemic to a particular place like malaria, urinary tract infection, typhoid needs to be performed. Radiological deterioration in Chest skiagram is a usual accompaniment in almost all cases of IRIS [153]. In some instances, increase in CD4 cell count may not be evident [156]. A decline of viral load less than 0.5 log10 copies/ml compared to baseline has a high negative predictive value in ruling out IRIS and is a mandatory criterion to differentiate it from HIV disease progression [159, 163]. The International network for the study of HIV associated IRIS (INSHI) criteria can be used for diagnosing unmasking IRIS and paradoxical IRIS [147]. Lipoarabinomannan (LAM) detection serves as a useful index of systemic antigen burden and has proved to be useful as a predictive marker [164].

When IRIS occurs at an extrapulmonary location, appropriate investigation can be performed and tissue specimen obtained wherever possible, and sent for bacteriological staining and culture in addition to routine histopathology. A negative culture that succeeds a pre-existing or baseline positive culture (that was sensitive to specific drug therapy) confirms the diagnosis of IRIS straightaway. However, this criteria of culture negativity cannot be applied for diagnosing unmasking IRIS or in paradoxical IRIS when the ATT-IRIS interval is too short. Hypercalcemia is a unique accompaniment of TB-IRIS, attributed to the calcium deposition through increased secretions of 1.25 dihydoxy cholecalciferol by activated macrophages and CD4 T cells [165].

### Differential diagnosis of IRIS

Drug resistant TB is the closest mimic that requires to be ruled out before steroids are administered as they form the cornerstone of IRIS therapy currently [166]. The phenomenon of “fall and rise” exhibited by the acid fast bacilli (described by Toman) typically mimics the temporal sequence of paradoxical IRIS [70]. Steroid administration in the face of MDR-TB could spell disaster, ending fatally. Zidovudine induced anemia also mimics IRIS presenting with fever, rigor that settles after appropriate modification of ART [88]. Late onset IRIS (occurring after 3 months of ART initiation) is not rare but re-estimation of viral load is compulsory in such patients, to rule out ART failure and HIV progression [167]. Lymphoma of the Non-Hodgkins type which could occur or co-exist with TB in HIV may flare up after ART and steroid administration could confuse the hispathology findings [168].

### Management

#### Preventive strategy

Intensive screening for TB and other opportunistic infections along with INH and cotrimaxozole prophylaxis reduces IRIS incidence [149, 167–169]. ART initiation before CD4 goes down considerably could protect against opportunistic infections and subsequent IRIS [148]. If a patient with TB has a CD4 > 50 cells/mm^3^, the initiation of ART can be delayed up to 8 weeks with close monitoring that reduces toxicity and IRIS but does not compromise on survival [169].

#### Treatment

Anti-inflammatory drugs especially steroids form the backbone therapy for TB-IRIS, even though non-steroidal anti-inflammatory agents could prove to be a useful initial therapy for milder and localized cases of IRIS [153, 170]. A dose of 0.5–2 mg/kg body weight is usually used and tapered in a 4–8 weeks period depending on the site and severity of inflammation. Premature withdrawal of steroids can cause recrudescence of symptoms [153]. Severe forms may require parenteral steroids initially followed by switch to oral steroids. Thalidomide in steroid dependent IRIS has shown good results. [171] Maraviroc, monteleukast, pentoxifylline have not been proved to be effective in IRIS treatment [172]. Experimental drugs for IRIS treatment in the pipeline include CXCR-3 antagonists, the main receptor for CXCL-10 [173], IL-18 binding protein and IL-6 inhibitors [174, 175], anti-CD28 therapy [176] and drugs modulating MMP-7 activity [158].

## Conclusions

HIV-associated pulmonary TB mandates a committed approach that encompasses both effective as well as enduring therapy originating from newer drug combinations, evolving ideas and emerging concepts from clinical trials globally, which if implemented in a proper and coordinated manner could not only save millions of lives but also offer a better quality of life to patients suffering from this coinfection.

## Abbreviations

HIV: human immune deficiency virus infection
TB: tuberculosis
PTB: pulmonary TB
ART: antiretroviral therapy
ATT: anti-tuberculosis therapy
LTBI: latent TB infection
TST: tuberculin sensitivity test
IGRA: interferon gamma releases assay
CXR: chest X-ray
AFB: acid fast bacillus
M.Tb: mycobacterium tuberculosis
MGIT: mycobacterial growth indicator tube
NAAT: nucleic acid amplification test
VEGF: vascular endothelial growth factor
ADA: adenosine deaminase
LAM: lipoarabinomannan
LPA: line probe assay
INH: isoniazid
IPT: INH preventive therapy
STCI: standards of TB care in India
DOT: directly observed therapy
ARR: acquired rifampicin resistance
NIRT: National Institute for Research in tuberculosis
CD: cluster of differentiation
MDR: multidrug resistant
XDR: extensively drug resistance
IRIS: immune reconstitution inflammatory syndrome
MMP: matrix metallo-proteinases
INSHI: international network for the study of HIV associated IRIS
